# Gradual shortness of breath

**DOI:** 10.4103/1817-1737.32236

**Published:** 2007

**Authors:** Jamal Al Deen Alkoteesh, Amer Shammas

**Affiliations:** *Radiology Department, Cork University Hospital, Wilton, Cork, Ireland*

A 46-year-old male presented via his puzzled general practitioner with complaint of a gradual progression of shortness of breath when exerting at work. He also had an annoying cough and was uncharacteristically tired. His only other complaint was of a sore red right eye that kept watering. On examination, the salivary glands were slightly swollen and tender.

A chest radiograph [Figure 1], CT scan [Figures 2 and 3] and gallium scan [Figure 4], shown here, were taken.

**Figure 1:**
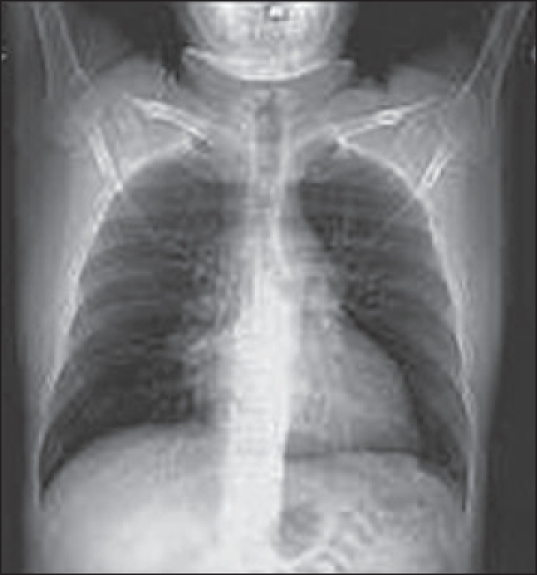
Chest X-ray

**Figure 2:**
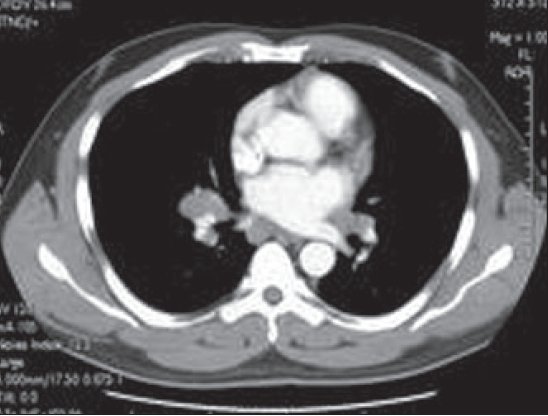
CT thorax with contrast

**Figure 3:**
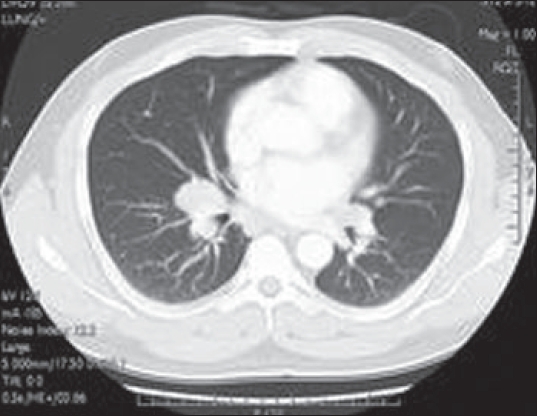
CT Thorax:lung window

**Figure 4:**
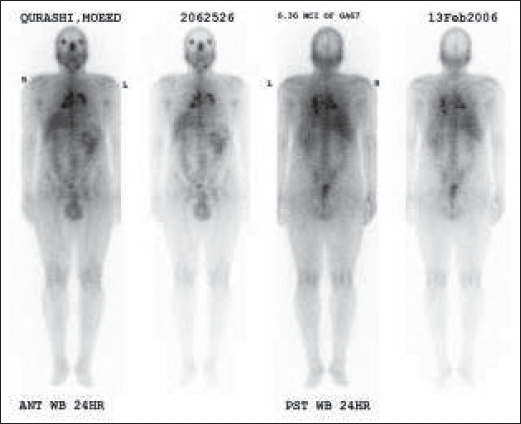
Galium scan

## Questions

- What radiological abnormalities are present on these images?
- What is your differential diagnosis for this abnormality?
- What other investigations might you consider to assist the diagnosis?

## Answers

The radiological abnormalities included bilateral hilar, anterior mediastinal, left para-aortic lymphadenopathy. There is no pulmonary infiltration. Gallium scan showed bilateral hilar nodal increased uptake and prominent uptake in the parotid, salivary and lacrimal glands (Panda sign). Lymphoma and sarcoidosis are the most likely differential diagnoses. Others to be considered are bronchial carcinoma (although likely to be unilateral) and TB.

The chest radiograph shows bilateral enlargement but does not show any features that allow a definitive diagnosis to be made. CT of the chest can help differentiate sarcoidosis from lymphoma; in lymphoma, the anterior mediastinal nodes are often enlarged. CT is also useful in showing the interstitial and bronchovascular inflammatory changes of sarcoidosis. Panda gallium uptake with bilateral symmetric lymphadenopathy is highly specific and considered characteristic. However, gallium 67 avidity alone cannot be used to establish a diagnosis of sarcoidosis. FDG-PET will show uptake within lymph nodes involved with sarcoid and extrathoracic lesions.

Biopsy, either core or fine needle aspiration, will often be necessary to differentiate the two conditions. If a superficial lymph node is found (in up to a third of patients), it may be conveniently biopsied. Otherwise, you may need to undertake a bronchoscopic, mediastinoscopic or CT-guided biopsy.

Other common areas and features of sarcoidosis are:

- Skin: erythema nodosum, lupus pernio, superficial lymphadenopathy
- Heart: cardiomyopathy, conduction abnormalities, pericardial effusion
- Eyes: uveitis (the complaint mentioned in this patient), keratoconjunctivitis sicca (dry eye syndrome)
- Spleen: splenomegaly
- Brain: granulomatous meningitis, brain infarct (from granulomas occluding small vessels)
- Kidney: hypercalcemic nephropathy
- Muscles and bones: dactylitis, arthralgia

## Discussion

The term sarcoidosis is derived from the Greek root ‘sarko,’ meaning flesh (as is the word sarcasm, which can be translated literally as ‘cut to the flesh’). The etiology and definitive treatment of this disease remain elusive.[1]

The chest disease can be classified into four stages for descriptive and prognostic purposes, as is shown in the table hereunder [Table 1].[2]

**Table 1:** Pulmonary sarcoidosis staging

Stage	Description
Stage 0	No radiographic abnormalities.
Stage 1	Hilar and mediastinal lymph node enlargement without pulmonary abnormalities. This combination occurs at some time during the disease in more than 90% of patients with sarcoidosis.
Stage 2	Hilar and mediastinal lymph node enlargement plus diffuse parenchymal disease.
Stage 3	Diffuse pulmonary disease unassociated with lymph node enlargement.
Stage 4	Diffuse fibrosis and end-stage lung might reasonably be categorized as Stage 4 disease.

Bilateral adenopathy is more common than right paratracheal or aortopulmonary window lymphadenopathy. There is often an inverse relationship between the adenopathy and the parenchymal disease. Types of parenchymal involvement include a reticular pattern, a miliary nodular pattern and a combination of these with larger nodules. The reticulo-nodular pattern is the most common parenchymal manifestation of disease.

The diagnosis of sarcoidosis can often be made with a considerable degree of certainty on a chest radiograph. The symmetry of the bilateral, hilar node enlargement along with the frequent associated enlargement of the right paratracheal and aortopulmonary window nodes is characteristic. The pulmonary parenchymal involvement is often symmetric also, and this is of diagnostic importance. The discrepancy between the extensive roentgen changes and the mild symptoms is the third finding of diagnostic significance. Finally, when interval roentgenograms are available, the process is typically observed to progress or regress slowly.[3]

Gallium lung scanning is positive in most patients who have sarcoidosis and may be observed in both intrathoracic and extrathoracic lymph nodes and in other organs. Gallium 67-avid disease has been reported in more than 90% of cases of pulmonary involvement. Gallium scan is more sensitive than a chest radiograph for detecting early disease. Characteristic patterns of uptake are seen in sarcoidosis. Early disease shows only bilateral hilar uptake. Bilateral hilar with paratracheal uptake is called Lambda sign. Prominent and symmetrical uptake in the nasopharyngeal region - parotid, salivary and lacrimal glands - has been referred to as Panda sign. Symmetrical bilateral hilar uptake with Panda sign has been reported to be highly specific for sarcoidosis. Patients with lymphoma usually have asymmetrical hilar uptake. Panda sign is not typically seen in lymphoma.[4]

Pulmonary accumulation of gallium in patients with sarcoidosis is associated with active disease (either alveolitis or granulomas). Classic radiographs do not necessarily represent progression or activity of the disease. Gallium scan provides the highest sensitivity for assessing disease activity when compared with chest radiography and serum ACE. In addition, negative gallium scans with normal serum ACE levels appear to have a high predictive value for excluding active sarcoidosis. Therefore, gallium scan appears to be valuable in follow-up patients who are undergoing therapy.[5]

Patients with sarcoidosis may present with a wide variety of symptoms and signs. The most common clinical manifestations include ocular disturbances, enlarged peripheral lymph nodes, skin eruptions and respiratory symptoms. A more common presentation in Europe, compared to the United States, is the syndrome of erythema nodosum with hilar lymph node enlargement, often associated with fever and arthralgia. The majority of patients are asymptomatic, however, and the disease is identified incidentally on chest radiography.[6]

The diagnosis can be made with confidence when tissue biopsy (typically of the scalene nodes or mediastinal biopsy) reveals noncaseating granuloma, especially with a positive Kveim test. The Kveim test consists of intradermal injection of 0.1 to 0.2 ml of crude saline suspension of sarcoid tissue, usually obtained from the spleen of patients with active sarcoidosis.

## References

[1] Paul LW, Juhl JH, Crummy AB (1987). Paul and Juhl's essentials of radiologic imaging.

[2] Paré JA, Fraser RG (1983). Synopsis of diseases of the chest.

[3] Sugie T, Hashimoto N, Iwai K (1994). Clinical and autopsy studies on prognosis of sarcoidosis. Nippon Rinsho.

[4] Sulavik SB, Spencer RP, Weed DA, Shapiro HR, Shiue ST, Castriotta RJ (1990). Recognition of distinctive patterns of gallium-67distribution in sarcoidosis. J Nucl Med.

[5] Johnson DG, Johnson SM, Harris CC, Piantadosi CA, Blinder RA, Coleman RE (1984). Ga-67 uptake in the lung in sarcoidosis. Radiology.

[6] Kohn H, Klech H, Mostbeck A, Kummer F (1982). 67Ga scanning for assessment of disease activity and therapy decisions in pulmonary sarcoidosis in comparison to chest radiography, serum ACE and blood T-lymphocytes. Eur J Nucl Med.

